# A high-throughput microfluidic diploid yeast long-term culturing (DYLC) chip capable of bud reorientation and concerted daughter dissection for replicative lifespan determination

**DOI:** 10.1186/s12951-022-01379-9

**Published:** 2022-03-31

**Authors:** Yingying Wang, Zhen Zhu, Ke Liu, Qin Xiao, Yangye Geng, Feng Xu, Shuiping Ouyang, Ke Zheng, Yimin Fan, Nan Jin, Xiangwei Zhao, Mario A. Marchisio, Dejing Pan, Qing-an Huang

**Affiliations:** 1 Key Laboratory of MEMS of Ministry of Education, Southeast University, Sipailou 2, Nanjing, 210096 China; 2 College of Chemical Engineering, Nanjing Forestry University, Longpan Road 159, Nanjing, 210037 China; 3 ZhongDa Hospital, Southeast University, Dingjiaqiao 87, Nanjing, 210009 China; 4 State Key Laboratory of Bioelectronics, Southeast University, Sipailou 2, Nanjing, 210096 China; 5 School of Pharmaceutical Science and Technology, Tianjin University, Weijin Road 92, Tianjin, 300072 China; 6 Cambridge-Suda Genomic Resource Center and Jiangsu Key Laboratory of Neuropsychiatric Diseases Research, Soochow University, Ren-ai Road 199, Suzhou, 215213 China

**Keywords:** Microfluidics, Single-cell analysis, *Saccharomyces cerevisiae*, Diploid yeast aging, Daughter-cell dissection, Replicative lifespan

## Abstract

**Background:**

Budding yeast, *Saccharomyces cerevisiae*, has been extensively favored as a model organism in aging and age-related studies, thanks to versatile microfluidic chips for cell dynamics assay and replicative lifespan (RLS) determination at single-cell resolution. However, previous microfluidic structures aiming to immobilize haploid yeast may impose excessive spatial constraint and mechanical stress on cells, especially for larger diploid cells that sprout in a bipolar pattern.

**Results:**

We developed a high-throughput microfluidic chip for diploid yeast long-term culturing (DYLC), optical inspection and cell-aging analysis. The DYLC chip features 1100 “leaky bowl”-shaped traps formatted in an array to dock single cells under laminar-perfused medium and effectively remove daughter cells by hydraulic shear forces. The delicate microstructures of cell traps enable hydrodynamic rotation of newborn buds, so as to ensure bud reorientation towards downstream and concerted daughter dissection thereafter. The traps provide sufficient space for cell-volume enlargement during aging, and thus properly alleviate structural compression and external stress on budding yeast. Trapping efficiency and long-term maintenance of single cells were optimized according to computational fluid dynamics simulations and experimental characterization in terms of critical parameters of the trap and array geometries. Owing to the self-filling of daughter cells dissected from traps upstream, an initial trapping efficiency of about 70% can rapidly reach a high value of over 92% after 4-hour cell culturing. During yeast proliferation and aging, cellular processes of growth, budding and daughter dissection were continuously tracked for over 60 h by time-lapse imaging. Yeast RLS and budding time interval (BTI) were directly calculated by the sequential two-digit codes indicating the budding status in images. With the employed diploid yeast strain, we obtained an RLS of 24.29 ± 3.65 generations, and verified the extension of BTI in the first couple of generations after birth and the last several generations approaching death, as well as cell de-synchronization along diploid yeast aging.

**Conclusions:**

The DYLC chip offers a promising platform for reliable capture and culturing of diploid yeast cells and for life-long tracking of cell dynamics and replicative aging processes so that grasping comprehensive insights of aging mechanism in complex eukaryotic cells.

**Graphical Abstract:**

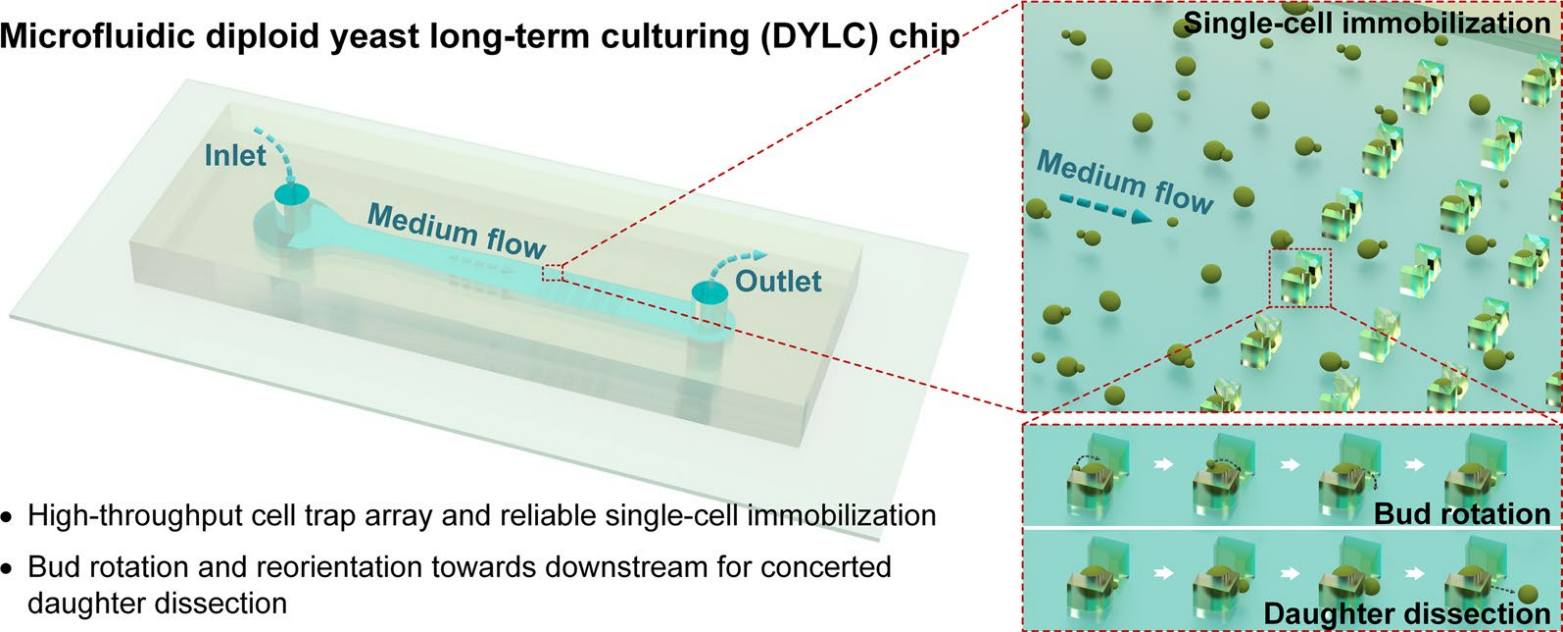

**Supplementary Information:**

The online version contains supplementary material available at 10.1186/s12951-022-01379-9.

## Background

Aging, along with age-associated health issues, has emerged as one of the vital research topics in life and medical sciences in recent years. Since 1959, budding yeast, *Saccharomyces cerevisiae*, has been brought insight in aging studies as a premier model cell owing to its rapid growth, short division time and low cost in harvest [1]. What’s more, budding yeast manifests convenience in facile genetic manipulation, and thus shows technical advantages in uncovering numerous signal pathways [2, 3] that link with abnormal lifespan and aging-induced pathology, such as neurodegeneration [4, 5] and cancer [6]. In aging and lifespan investigations with budding yeast cells, haploids are typically used to study genomic regulation and physiological functions [7, 8], while diploids, with two sets of genomes could show distinguished functions in complementary aging assays, such as intrinsic and extrinsic noise studies [9, 10].

In yeast aging, the replicative lifespan (RLS) is defined as the number of daughters mitotically produced by a mother cell. As daughter cells usually aggregate around their mother cell to form a cluster, the instantaneous removal of daughter cells was considered of great necessity for continuous mother-cell tracking and RLS determination. Traditionally, RLS was obtained by manually removing daughter cells with a microneedle under a microscope and counting mature progenies until the mother cell died [1]. However, this method is low-throughput, labor-intensive and time-consuming [11]. Additionally, artificial bias could be introduced in RLS determination in practical operation, such as the temperature fluctuations during the repeated refrigeration of yeast culture plates overnight and the potential cell damage during the manual separation of yeast mothers and daughters.

With the rapid development of microtechnology and biosensors, microfluidic systems for budding yeast culturing and aging studies have substituted the conventional approaches because of high-throughput, automated and precise micromanipulation of single yeast cells, along with optical inspection. In microfluidic devices, firstly, a large number of samples can be obtained from one single running of yeast cell culturing. Second, immobilized single cells undergo automated daughter dissections by fluidic shearing forces. Third, continuous medium perfusion with precisely controlled microenvironment enables continuous dissection of daughter cells, thereby greatly reducing the time consumption of experiments from 4 weeks in conventional methods to 3 days in microfluidic devices. Previously, three typical microstructures were designed to accommodate budding yeast cells, which could be summarized as “pensile pads”, “column traps” and “daughter collectors”. (i) The “pensile pads” were polydimethylsiloxane (PDMS) microstructures extended from the channel ceiling close to the substrate leaving a proper gap in between. Cells were first loaded with a high pressure, pushing the pads upwards. Then, the pressure was released suddenly, leaving mother cells clamped underneath the pads and smaller daughter cells washed away by continuous medium perfusion [12, 13]. (ii) The “column traps” are array-formatted micropillars, each of which, as an elementary unit for single-cell immobilization and culturing, features two baffles forming a wider opening upstream and a narrower orifice downstream to stop and dock the bypassing yeast cells in suspension. The cell-trap structures though differing in geometry, e.g., “/ \” shape [14] and “U” shape [15–17], were essentially designed to retain mother cells by pushing them inside the traps, and leave their progenies forming outside and subsequently being removed by hydrodynamic shear forces. (iii) The “daughter collectors” are cell-trap structures such as tri-post cages [16] or “[ ]” -shaped cages [18], which initially stopped yeast cells outside, then collected their first daughters budded into traps, and afterwards cultured the virgin daughter cells by continuous dissection of their mature buds.

Concerning reliable immobilization, long-term culturing and concerted daughter-dissection of single diploid yeast cells that bear significant differences in terms of cell size and budding pattern, the aforementioned microfluidic chips, mostly for culturing haploid yeast, were not desirable to diploids. First, limited spaces in previous cell-trap microstructures for haploid cells (4-µm spheroids) are not capable to accommodate larger diploid yeast cells (5 × 6-µm ellipsoids), which not only require larger space in traps but also undergo twice volume enlargement during aging process [18]. Moreover, aged yeast with auxesis deformed under the compression in the microstructures, such as “pensile pads” [12, 13]and tri-post traps [16], leaving the uncertain influence on cell aging. Second, daughter dissection in previous cell traps, such as the “/ \”-shaped [14], “U”-shaped [15] and tri-post traps [16], was happened in disorder, namely that daughters were removed upstream or downstream, highly depending on the original bud sites when trapping the mother cells. Since haploid cells bud in an axial pattern (i.e., forming new buds adjacent to the previous bud site) [19], their daughters could be always dissected in the same position. By contrast, diploid cells bud in a bipolar pattern (i.e., forming new buds either adjacent or opposite to the previous bud site) [20, 21], and even bud randomly at a higher frequency as they age [22]. Daughters of diploids could then be formed in all directions throughout their lifespans. As a result, the random position for daughter dissection in previous traps may exert different hydrodynamic forces on cells, which may influence the stability of long-term cell maintenance and the repeatability of RLS determination. Therefore, new design of microfluidic devices that allow for reliable mother maintenance and concerted daughter dissection of diploid yeast are in great desire.

In our previous work, several microfluidic devices with cell traps featuring 20 to 30-µm-high and 3-µm-wide orifices have been developed to capture and monitor single diploid yeast over an extended time [23, 24]. Interestingly, cells immobilized at the traps could rotate and then reorientate their buds towards the low-pressure side downstream, which inspired us that elongated cell-trap microstructures instead of compressed ones could provide free space for cell rotation so that potentially capable of daughter dissection in a concerted position.

In this work, we reported on a high-throughput microfluidic chip for diploid yeast long-term culturing (DYLC), time-lapse imaging, cell-aging analysis and RLS determination. The DYLC chip was patterned with 1100 “leaky bowl”-shaped traps formatted in an array to accommodate single yeast cells and remove daughters under laminar-perfused medium. The delicate microstructures of cell traps featured bud rotation and reorientation towards downstream and concerted daughter dissection by hydrodynamic forces. Finite element modeling (FEM) and computational fluid dynamics (CFD) simulations were performed to optimize the geometric settings of the cell-trap array. Critical parameters of cell traps were then characterized in experiments not only to ensure long-term reliable retention of mother cells and bud rotation for concerted daughter dissection, but also to provide sufficient space for volume enlargement of diploid cells during growth. Afterwards, continuous culturing and time-lapse monitoring of diploid yeast cells immobilized in the DYLC chip were carried out for over 60 h, followed by off-line image analysis to determine RLS and budding time interval (BTI) during yeast aging.

## Results

### Design concept

**Fig. 1:**
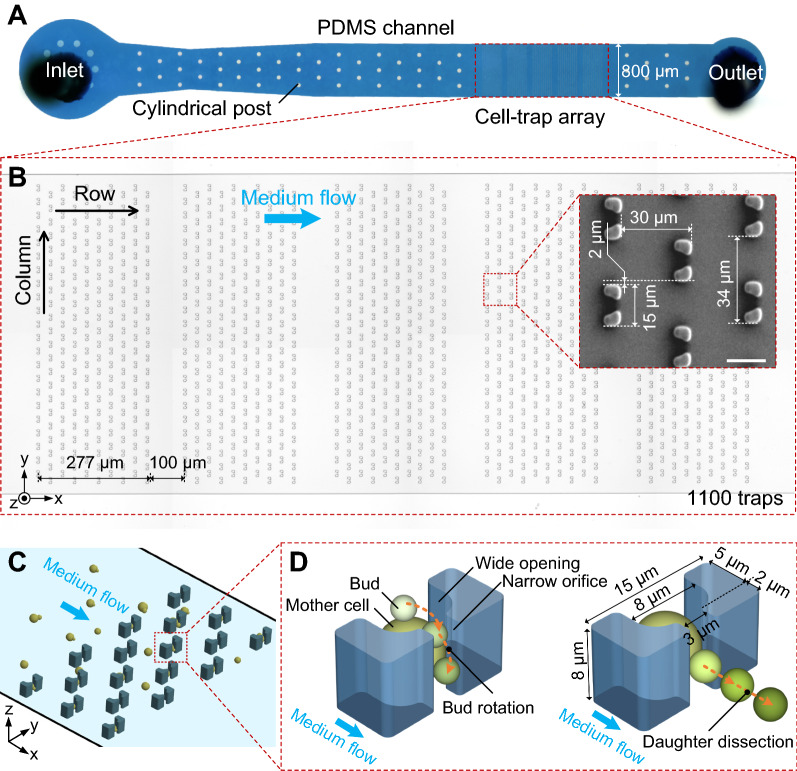
Overview of the microfluidic DYLC chip. **A** Photograph of the PDMS microchannel including 53 cylindrical posts and a cell-trap array. **B** Micrograph of the array patterning into 5 subarrays with a gap of 100 μm in between. Each subarray contains 10 × 22 traps. Distances between columns and rows are 30 and 34 μm, respectively. Traps are aligned to the middle of the spacings between two neighboring traps in adjacent columns. Insert is a SEM micrograph of the trap array. Scale bar is 10 μm. **C** A schematic cartoon showing the cell loading and trapping processes in the array. **D** 3D schematics showing the geometric dimension of the “leaky bowl”-shaped trap, the hydrodynamic bud rotation and the concerted daughter dissection. The trap features a 7 μm × 15 μm × 8 μm outer profile in xyz-dimension, with a 5 μm × 8 μm wide opening upstream and a 2 μm × 3 μm narrow orifice downstream. The bud of immobilized mother cell tends to be rotated into the narrow opening downstream and then removed under the hydrodynamic shearing forces

The high-throughput microfluidic DYLC chip was designed to capture single budding yeast cells, reliably retain mother cells, and effectively remove daughter cells so as to perform long-term culturing and microscopic monitoring for later-on image analysis to determine yeast RLS. Figure 1 shows the micrographs and schematics of the microfluidic chip, which simply consists of an 8-µm-high PDMS microchannel bonded on a glass substrate. The microchannel features an inlet for cell suspension and medium infusion, 53 cylindrical posts, 1100 “leaky bowl”-shaped traps formatted in an array, and an outlet for waste collection (Fig. 1A). The posts, with the diameter of 30 μm, were designed to support the ceiling of the wide microfluidic channel from collapse during chip bonding. In order to prevent the whole channel clogging among the dense traps, the trapping array was patterned into 5 subarrays with a spacing of 100 μm in between (Fig. 1B). Each subarray contains 220 traps (10 rows × 22 columns) with optimized distances of 30 and 34 μm between adjacent columns and rows, respectively. Traps in each column are aligned to the middle of spacings between two neighboring traps in adjacent columns. Such setting of the trapping array ensures both efficient trapping and enough growing space of yeast cells. Each trap (outer profile: 15 μm × 7 μm) comprises two pillars facing each other, together forming a “bowl”-shaped wide opening (width: 8 μm) upstream and a “leaky” orifice (width: 3 μm) downstream. The length of the wide opening was optimized to 5 μm to ensure the yeast trapping in single-cell resolution, and the length of the orifice was set to 2 μm to minimize the structural squeezing or mechanical stress on newborn buds during their growth. Once yeast suspension was infused into the microchannel, cells were dragged to be docked inside the empty “bowls” by hydrodynamic forces (Fig. 1C). Since the height (8 μm) of each trap is larger than the diameter of budding yeast cells (4 ~ 6 μm), immobilized single cells with small buds have sufficient space vertically to rotate in the “bowls” under hydrodynamic forces, and buds could be ultimately orientated and clamped in the orifice downstream when growing bigger (Fig. 1D). After the completion of cytokinesis, the matured buds (i.e., daughter cells) could be automatically detached by hydrodynamic shearing forces whereas mother cells were stably retained in their traps. Therefore, the immobilized yeast cells during their entire lifespans could undergo consistent hydraulic microenvironments, such as the hydrodynamic forces in cell growth, mother retention and daughter removal. In particular, for accurate RLS determination with the diploid budding yeast, which features random budding spots on mother cells, the delicate “leaky bowl”-shaped traps with the optimal dimensions could thus provide the merits of free mother rotation in the traps, bud reorientation to the narrow orifices and concerted daughter dissection.

### CFD simulation of fluid field in the cell-trap array

**Fig. 2:**
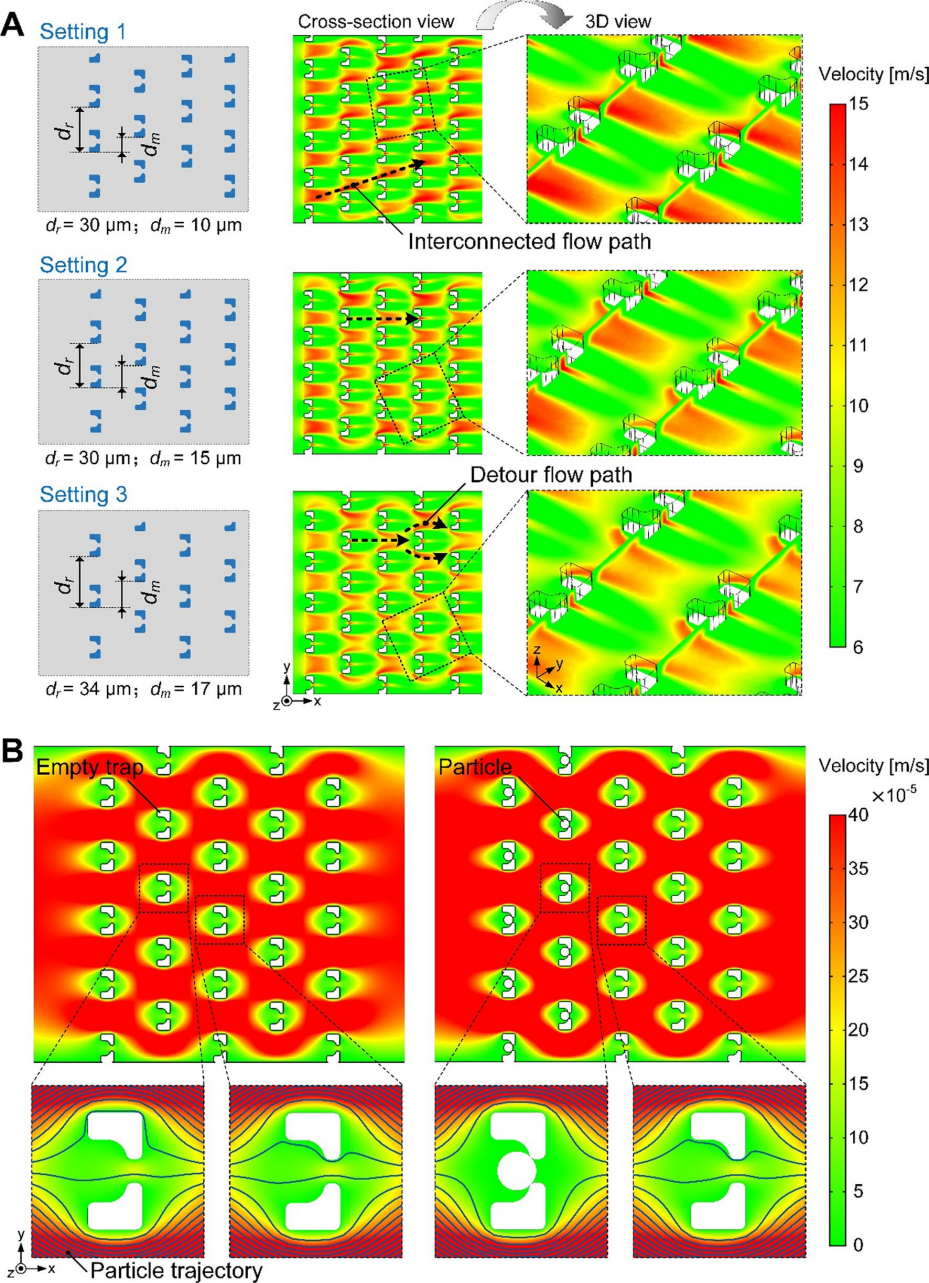
CFD simulations of fluid field in a 5 × 5 cell-trap array. **A** Flow velocity distribution across the xy-plane (4 μm above the bottom) of the array with three geometric settings (*d*_*r*_: 30 μm, 30 and 34 μm; and *d*_*m*_: 10 μm, 15 and 17 μm, respectively). **B** Particle trajectory tracing before and after cell immobilization in the traps upstream. Trajectories indicate the flow path of cells during the loading process. The array was set to 34 μm (*d*_*r*_) and 17 μm (*d*_*m*_)

To better understand the cell-trapping process and therefore optimize the geometric arrangement of cell traps, the fluid field distribution in the array was calculated in CFD simulation under three different settings of the distance between adjacent rows (*d*_*r*_) and the misalignment in rows (*d*_*m*_). Contour plots in Fig. 2A show the flow velocity distributions across the 5 × 5 cell-trap array in the xy-plane. Compared to the flow velocity distribution in setting 2, the region with high value of flow velocity in setting 1 is almost interconnected so that cells are prone to bypass traps and flow along this region towards downstream. In setting 2, dissected daughters from upstream tend to be captured by traps downstream, while in setting 3 they are inclined to make a detour around traps downstream due to larger *d*_*r*_. Moreover, for practical considerations, slightly larger *d*_*r*_ in setting 3 could reduce the occurrence of channel clogging caused by abnormally large cells when approaching apoptosis. Hence, the distance between adjacent rows and the misalignment in rows of the cell-trap array were suggested to be 34 μm and 17 μm, respectively.

With the suggested geometric setting of the array, particle trajectory tracing was then simulated to investigate the process of cell trapping before and after the traps upstream were docked with cells (Fig. 2B). Due to the low flow resistance through the orifices of the “leaky bowl”-shaped traps, suspended cells could be initially dragged towards the empty traps under hydrodynamic forces. Once the wide openings were occupied, drastic increase of flow resistance in traps rendered subsequent cells bypassing these traps and flowing towards the empty traps downstream (Additional file 2: Video S1). Such dynamic process of single-cell trapping was observed in experiments (Additional file 3: Video S2). A budded cell, rotating with the medium stream, was immobilized in an empty trap, and then cell suspension from upstream bypassed this occupied trap successfully. Therefore, the simulation results demonstrated that cells in the loading process could rapidly fill the array by evading the occupied traps and giving preference for empty ones.

### Optimization of trapping efficiency and long-term maintenance of budding yeast cells

**Fig. 3:**
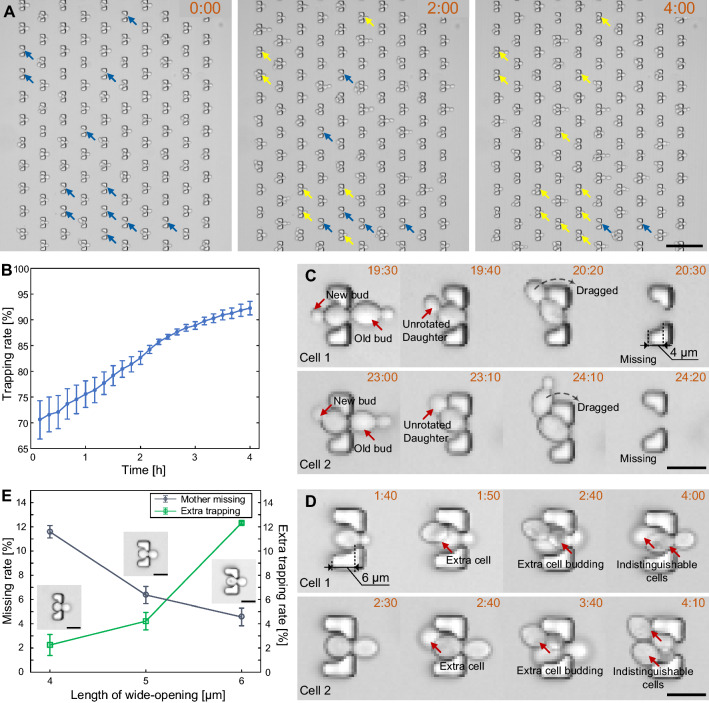
Optimization of trapping efficiency and long-term maintenance of budding yeast cells. **A** Time-lapse images showing the filling of empty traps at 0 h, 2 and 4 h after cell loading (time mark: “hour: minute”, the same below). Blue arrows indicate empty traps, while yellow ones indicate traps that were docked with daughter cells from upstream. Scale bar is 50 μm. **B** Trapping rate increasing within the first 4 h after cell loading (3 independent runs). **C** Two samples of mother cells being dragged away by unrotated buds in the 4-µm-long “bowl”. Scale bar is 10 μm. **D** Two samples of capturing extra cells in the 6-µm-long “bowl”. Scale bar is 10 μm. **E** Percentage of occupied traps occurring with mother loss and extra cell capture from 0 to 4 h and from 24 to 28 h after cell loading (3 independent runs, 220 traps per run). Scale bar is 10 μm

The efficiency and reliability of single-cell immobilization plays a vital role in sample collection for high-throughput imaging and data analysis. With the optimal design (*d*_*r*_ = 34 μm and *d*_*m*_ = 17 μm) of the cell-trap array, the cell-trapping efficiency was investigated in experiment. After cell loading, an initial trapping rate could reach 70.5% on average. The trapping rate could be raised by increasing the concentration of cell suspension and the duration of cell loading. However, a higher trapping efficiency in the beginning of the experiments was not demanded since empty traps could be filled with daughter cells removed from traps upstream in the next several hours during cell culturing (Fig. 3A). Within the first 4 h of yeast culturing, the cell-trapping efficiency could be raised from 70.5 to 92.3% (Fig. 3B), thereby providing a high-throughput platform for yeast immobilization. With the ability of self-filling among the staggered traps, low concentration of cell suspension was preferred for single-cell immobilization.

Next, we investigated the capability of single-cell maintenance in traps that differed in the length of the “bowl”-shaped wide opening (i.e., 4 μm, 5 and 6 μm). In the experiment with 4-µm-long “bowl” of traps, large mother cells, when approaching apoptosis, could be dragged away by unrotated buds that were located upstream (Fig. 3C). Such loss of mother cells could dramatically cut down the number of samples for RLS determination. In the experiment with 6-µm-long “bowls”, which provide sufficient space to dock two small yeast cells, traps occupied with single yeast cells could capture extra cells coming from the upstream (Fig. 3D). The extra cell trapping could affect the imaging quality and identification of original mother cells in long-term cell culturing. Figure 3E shows the percentages of occupied traps that were occurred with mother loss and extra cell capture during 0 to 4 h and 24 to 28 h after cell loading. We can see that the mother-missing rate gradually decreases with the increase of “bowl” length, while the extra-trapping rate rises up. Therefore, the length of wide-opening was optimized to 5 μm for the sake of stability in single-cell retention.

### CFD simulation and experimental characterization of bud rotation for concerted daughter dissection

A haploid budding yeast always forms new buds adjacent to the scars of previous daughters [19, 20]. Thus, haploid yeast that initially sprouted downstream in the microfluidic traps could undergo successive removal of daughters in following generations [14, 15]. However, diploid budding yeasts, which forms new buds either adjacent or opposite to the previous bud site, would have high risks of being dragged away when buds are towards the upstream of traps. Hence, the microfluidic DYLC chip was designed to ensure concerted daughter removal of diploid budding yeast cells within consecutive generations by taking the advantages of bud reorientation into the unified orifices downstream.

Firstly, bud reorientation of immobilized budding yeast under continuous fluidic perfusion was theoretically analyzed by numerical simulation of hydrodynamic forces on a single cell docked in a trap (Additional file 1: Fig. S1A). When the bud was set to 0 (i.e., along -x direction) or 45 degree, the hydrodynamic force on bud points obliquely upwards, showing the motional tendency of the bud. After the bud rotated over 90 degree (i.e., along + z direction), the force points obliquely downwards, indicating the bud to be orientated into the orifice. At 180 degree (i.e., along + x direction), the z-component of the hydrodynamic force on the bud is negative, meaning that the reoriented bud could be stably maintained in the orifice. Therefore, the simulation result shows the feasibility of bud rotation from upstream to downstream in the orifice under hydrodynamic forces. Moreover, with the increase of bud diameter from 2 to 4 μm, the x-component of hydrodynamic forces gradually rises up and dominates during the bud growth (Figure S1B), suggesting that larger bud could be retained in the orifice more stably till the consequent daughter dissection had occurred after the completion of cytokinesis. Also, larger bud possesses higher hydrodynamic force for dragging the bud downstream to accomplish daughter detachment.

**Fig. 4:**
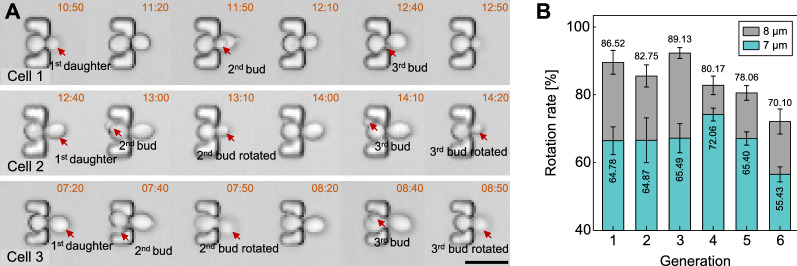
Characterization of yeast cell rotation and but reorientation in traps. **A** Time-lapse images showing the rotation of newborn buds, which were sprouted in adjacent, opposite and random spots regarding previous ones in three consecutive generations. Trap height is 8 μm. Scale bar is 10 μm. **B** Cell rotation rate in the initial 6 generations. Cells were immobilized in traps with the height of 7 μm versus 8 μm (3 independent runs, 220 traps per run)

Secondly, occurrences of bud rotation in experiment were verified by time-lapse images, especially with the mother cells that had two buds during a certain time period (Fig. 4A). In the case of Cell 1, the small bud (2nd bud) sprouted next to the unseparated daughter (1st daughter), and the 2nd bud remained in the narrow orifice downstream after the separation of 1st daughter. The 3rd bud successfully oriented into the orifice following the same path as the 2nd one did. The cases of Cell 2 and Cell 3 demonstrate the reorientation of new buds that sprouted on opposite and random sites of previous daughters, respectively. We could clearly observe that newborn buds (2nd buds) appeared before the removal of daughters (1st daughters). In the next images, the buds were oriented into the orifice downstream and meanwhile the mature daughters disappeared. Afterwards, the buds remained downstream throughout their division periods, followed by their dissection and the reorientation of new progenies (3rd buds) into the orifice. Furthermore, Videos S3 and S4 show the dynamic rotation and reorientation of immobilized budding yeast cell. Initially, the mother cell with a tiny bud was freely rotating in the “bowl”-shaped trap. With the bud growth, the cell stopped rotating with its bud clamped in the narrow orifice and swayed under the continuous medium perfusion. As the bud grew larger, the bud became motionless until it was dissected after the completion of cytokinesis (Additional file 4: Video S3). Right after the removal of the mature progeny, the following bud was immediately rotated into to the orifice (Additional file 5: Video S4).

Thirdly, the rotation rates of cells immobilized in two trap arrays that differed in height (7 μm vs. 8 μm) were compared in terms of the initial six generations of buds. The percentage of successful reorientation towards the downstream in traps with the height of 7 and 8 μm were 64.67% and 81.12%, respectively (Fig. 4B). The result indicated that 8-µm-high traps provided sufficient space for cell rotation and consequently concerted daughter dissection. Video S5 recorded the whole lifespan of an immobilized budding yeast cell. We could observe the successful orientation of buds towards the orifice downstream followed by concerted daughter dissection in the orifice for the 25 generations. However, bud rotation occurred less as the mother cell aged. This phenomenon could be attributed to following reasons: One is that the size increase of the senescent mother severely restricted the mobility of buds in traps; the other one is that the delayed detachment of daughter cells rendered the new buds growing too large to be rotated.

Therefore, the above experimental results demonstrated that the “leaky bowl”-shaped yeast traps enable efficient cell rotation by hydrodynamic forces, and cells could subsequently undergo the concerted processes of bud reorientation and daughter dissection in successive division cycles until apoptosis.

### High-throughput RLS determination of diploid budding yeast

**Fig. 5:**
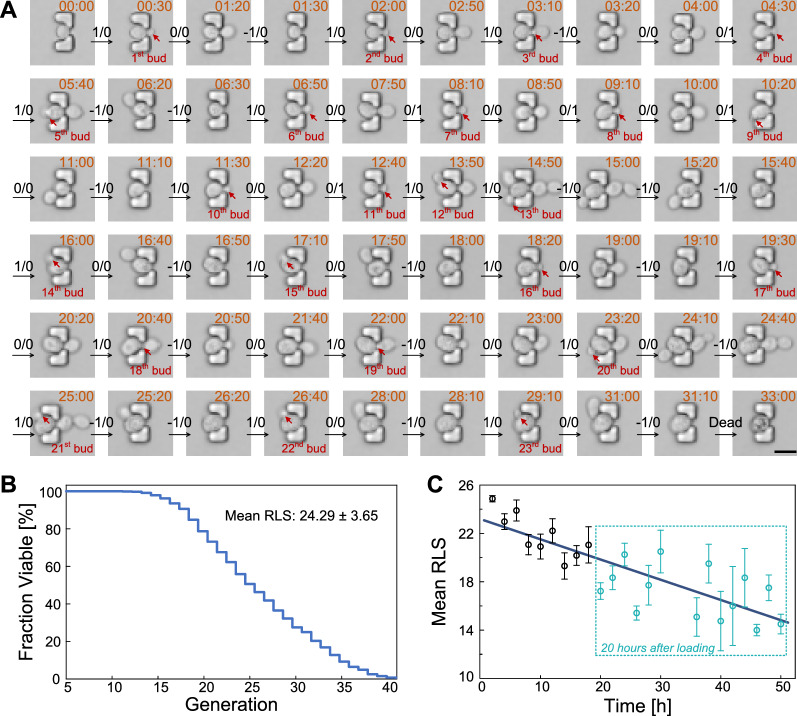
Long-term monitoring and RLS determination of diploid budding yeast cells in the DYLC chip. **A** Time-lapse images of a representative budding yeast cell in aging. Sequential two-digit codes indicate the bud appearance and daughter dissection. Scale bar is 10 μm. **B** Cell viability plotted as a function of the number of generations (n = 786 in 3 independent runs). **C** Mean RLS of cells captured within every 2 h. The dark blue line indicates a linear regression of the correlation between the mean RLS and the time of capturing cells (n = 997 cells in 3 independent runs)

To validate the capability and reliability of the microfluidic DYLC chip in high-throughput aging study of diploid budding yeast cells, long-term monitoring of single immobilized cells for over 60 h was performed. Figure 5A shows one representative cell that continuously gave birth to new buds until its death. The corresponding data analysis of this cell was conducted to enumerate the budding and dissection events throughout its lifespan by using the sequential two-digit coding. RLS of this cell, 23 generations, was calculated from the recorded digital matrix and its converted oscillogram (Additional file 1: Fig. S2). According to the data analysis of 786 cells trapped in the initial 10 h of 3 independent experiments, we obtained the reduction in cell viability as a function of replicative ages (Fig. 5B), resulting in an average RLS of 24.29 ± 3.65 generations. Then, additional 211 cells captured after the first 10-hour running of experiments were included in RLS analysis. We redistributed all 997 cells by grouping them according to the time point of the mother-cell trapping at a 2-hour interval (Fig. 5C). The fitting curve shows statistically shortened lifespans through the whole data set. The mean RLS (24.87 generations) was highest at the first 2 h of the experiments, while the minimum (only 14.25 generations) appeared on cells captured between 44 and 46 h. Moreover, the mean RLS of cells captured after 20-hour running was 17.21, a large decrease of 29.15% compared to that in the first 10 h. This phenomenon implies that a portion of senescent mother cells after 20-hour culturing may produce daughters that could not inherit full lifespan [25]. Due to the asymmetric division between mother and daughter yeast, the shortened lifespan in daughters from old mothers has been debated at the protein level. The function of asymmetric distribution of proteins, which accumulate steadily as mother cells aged, usually keep the protein level in daughter cells lower than that in their mothers. However, as was proposed and verified that daughters born from old mother cells have a protein level comparable to middle aged mothers, the lifespan could be limited when the inherited proteins reach a proper level [26]. The downward trend of the linear fitting curve in Fig. 5C also agrees well with the reported results that lifespan reduction in daughters may accumulate progressively as the age of their mothers increased.

### Budding time interval (BTI) of diploid yeast cells throughout the lifespan

**Fig. 6:**
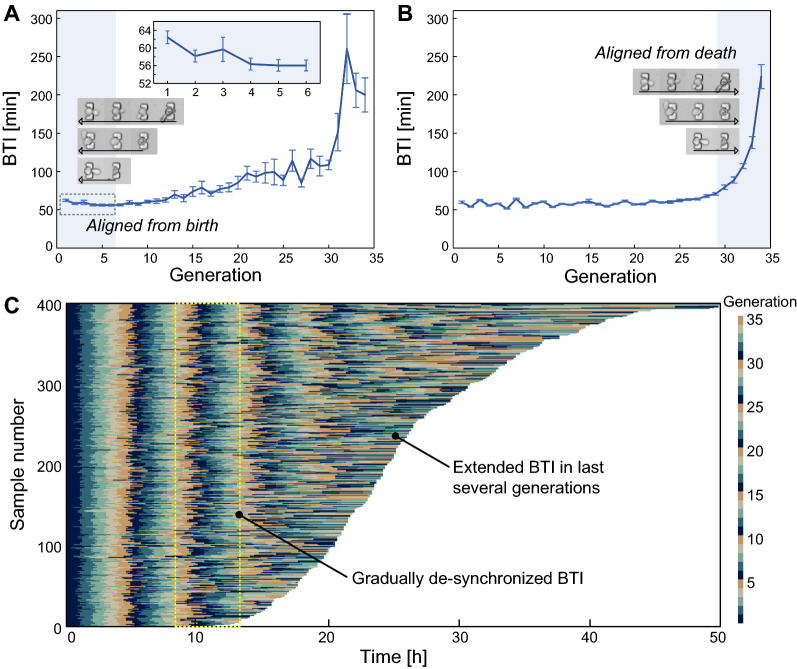
Budding time interval (BTI) of diploid yeast cells in replicative aging. **A** Mean BTI with all samples aligned to birth (n = 33). **B** Mean BTI with all samples aligned to death (n = 33). **C** Kymograph of BTI. In every 5 generations, each BTI was marked with a distinctive color, for better illustration of the synchronization and de-synchronization among individual cells. Samples were ordered by their RLS (n = 400)

The microfluidic DYLC chip enables large scale screening of cell samples to investigate the age-related changes in BTI. Since the time duration for the first G1 phase of haploid yeast fluctuated over a tenfold range, the mean budding time interval (BTI) of the first generation of haploid yeast could show great extension than subsequent generations [27]. To investigate the dynamic BTI of diploid budding yeast cells at birth, 33 mother cells in a small size and initially showing no obvious bud were selected and analyzed. The budding time interval (BTI) was obtained from the digital matrix (Additional file 1: Fig. S2). As illustrated in Fig. 6A, mean BTI of the first generation of diploid cells exhibits an extension of 9%, compared to that of the next five generations, similar to the haploid yeast. To investigate the BTI variation upon cell apoptosis, BTI of the 33 mother cells was then calculated in death-centric perspective by aligning all data to the last generation in their lifespan. The result in Fig. 6B demonstrates that BTI of diploid yeast cells is almost steady during most of the lifespan, whereas it rises up dramatically in the last several generations.

It is commonly recognized that the stochasticity and robustness are inherent properties of living cells [28]. However, experimental results derived from conventional approaches were usually based on assumptions that cells performed uniform growth and division and the group behaviors could hide heterogeneities among individual cells. The cellular de-synchronization in diploid yeast aging was verified through 400 samples from 3 independent experiments, where cells were relatively small in size and with no obvious bud initially. For better visualization, BTIs in every 5 consecutive generations were periodically marked with 5 different colors in a kymograph. As shown in Fig. 6C, the BTI distribution remains synchronized within the first 8 to 10 generations, whereas it gradually differentiated and elongated as cells progressively approached to the end of their lives. The BTI divergence may demonstrate the heterogeneity among individual cells even under the same microenvironment. In addition, the result of diploid yeast cells may exhibit better stability than haploids, which started to de-synchronize after about 5 divisions [14]. Therefore, the consistence of the mean BTI and BTI distribution until the last few generations indicates that diploid yeast cells have a better resistance to extrinsic fluctuations and intrinsic noises, such as environmental stress, protein accumulation and DNA damage during their lifespans [29].

## Discussion

Investigations on diploid budding yeast cells, which harbor allelic information such as dominant and recessive genes, become a significant step towards further understanding of complicated phenotypes of aging and thus promote the establishment of profound aging theories on eukaryotes. Previous endeavors on yeast RLS determination performed with either micromanipulation or microfluidic devices have rarely focused on diploids, leaving large blanks in the field of aging studies.

This work has presented a high-throughput microfluidic DYLC chip with 1100 arrayed-formatted and “leaky bowl”-shaped traps, which are capable of reliable immobilization, long-term culturing, optical inspection and replicative aging analysis of single diploid budding yeast cells. Each “leaky bowl”-shaped trap, 8 μm in height, has been structured with a 5-µm × 8-µm (length × width) wide opening upstream for reliable retention and free rotation of diploid yeast mothers, and a 2-µm × 3-µm narrow orifice downstream for continuous growth and ultimate dissection of daughters. With the optimization of array settings in row and column spacing, the chip could reach 9.3% trapping efficiency of single cells in the first 4-hour culturing. Immobilized cells in traps could then undergo consistent hydrodynamic microenvironments, which, associated with the delicate “leaky bowl” microstructures, have induced cell rotation, bud reorientation towards the narrow orifice downstream and concerted daughter dissection. As such, the microfluidic DYLC chip has been successfully applied to long-term single-cell culturing for over 60 h, automated progeny removal at concerted positions and time-lapse tracking of diploid yeast replicative aging process. In off-line image analysis, RLS and BTI have been calculated by using sequential two-digit coding to mark the change of bud number, which corresponded to the cellular events of budding and daughter dissection. The mean BTI showed slight extension in the first generation, stability at middle age and drastic extension in the last few generations. Such variation of BTI distribution in single-cell level evidenced the stochastic cell process. Compared to haploids, the delayed de-synchronization in diploids have shown better stability in regulation of cell replication. Experimental results have demonstrated that the microfluidic DYLC chip is a promising platform for high-throughput and long-term yeast culturing, RLS determination and cell dynamics investigation of diploid budding yeast cells.

In comparison with previous microfluidic devices for budding yeast culturing and replicative aging studies, the microfluidic DYLC chip offers several advantages. (i) The chip shows quickness, convenience and simplicity in fabrication and operation. With standard PDMS-based soft-lithography process, dozens of chips could be assembled within 3 h using the pre-patterned SU-8 master. The loading and capturing processes of cell suspension are simply done within 5 min. (ii) A large number of available samples can be obtained from one independent running of long-term diploid yeast culturing in the microfluidic DYLC chip. Furthermore, the throughput of cell samples could be increased by simply bonding more parallel channels on the same glass substrate in one fabrication step. (iii) The geometrical settings of “leaky bowl” traps, i.e., the elongated height, wide openings and narrow orifices, are properly optimized to provide enough space for the volume enlargement of diploid cells throughout the whole lifespan. In this circumstance, the structural compression exerting on both mothers and daughters are reduced, so as to alleviate mechanical stress on cells in aging. (iv) The innovative microstructures of cell traps ensure newborn buds rotating and reorientating to narrow orifices downstream under the laminar-perfused medium, so as to induce concerted dissection of daughters by hydrodynamic forces. Such design for cell rotation, bud reorientation and daughter removal overcome the bipolar budding pattern of diploid yeast cells, which may form new buds upstream in traps and may be dragged away by buds growing upstream, thereby meeting the demands of reliable retention of diploid yeast cells.

For the replicative aging of diploid budding yeast, an RLS value of 24.29 ± 3.65 generations was obtained from 786 cells in 3 independent experiments. This value is consistent with the reported range of diploid yeast, but in a relatively lower level, as summarized in Table S1. However, the RLS values vary considerably among different publications. The diploid yeast has a replicative age from 24 to 37.5 generations and the haploid age varies from 21 to 29.96 generations. Such variation could be attributed to the method of daughter dissections, the manual selection of samples, the design of microfluidic structures, and the data processing algorithm of RLS calculation. (i) Manual dissections result in longer RLS than those from microfluidic devices in most experiments. It might be due to the artificial bias in data selection by operators. For instance, cells on the plates that showed 40% decrease of RLS than other plates were discarded [30], and mother cells that produced less than 5 daughters were abandoned [31]. (ii) Different microfluidic structures for cell retention and daughter dissections may generate different mechanical stress or shearing force, thereby affecting cell aging and RLS values. For example, the “pensile pads” structures showed shorter lifespan of haploid yeast cells, compared to the values from “columns traps” [32]. (iii) Data processing algorithms may also result in variation of RLS values. The mean RLS of haploid yeast increased from 22.4 generations (original data) to 24.7 (compensated data by using Kaplan-Meier estimator) [14]. In comparison, with the DYLC chip and the image analysis method, we reported the original RLS values of diploid budding yeast cells without any sample filtering or data compensation. In addition, cell samples that were dissected from senescent mothers and immobilized, were also used in the RLS determination, which may contribute to the average lifespan at a relatively lower level among the presented values.

The present DYLC chip for diploid yeast culturing and replicative aging analysis still requires further development to improve its performance. (i) The occurrence of bud rotation diminished as mother cells aged. This phenomenon led to considerable number of missed samples of old mothers since they were dragged away by upstream grown daughters. (ii) Dissected daughter cells flowing from upstream could soon dock in the traps where the former mother cells were dragged away. The missing of old samples and trapping of new samples may occur between two adjacent images and thus affected the accurate identification of cell budding status in RLS and BTI calculation. To eliminate such misleading samples, attentive screening and data review were highly required during image analysis.

## Conclusions

In short, the proposed microfluidic DYLC chip enables high-throughput capturing, reliable maintenance and long-term culturing of diploid yeast cells, as well as RLS determination and dynamic morphological analysis in yeast aging studies. Therefore, the chip opens up potentials of complete relevance tracing between replicative lifespan and age-associated morphologic variations, including cell-cycle duration, cell volume and growth rate. In addition, as the diploid budding yeast cells possess a pair of complementary chromosomes, the microfluidic DYLC chip could shed light on complex age-associated regulations studies such as loss of heterozygosity (LOH) [33], DNA repairment in mating and silent information regulation [34]. Combined with fluorescent observation, we envision that the DYLC chip could study phenotypes and protein localization of both homogeneous performance of cell populations and heterogeneous behavior of individual single cells.

## Methods

### Chip fabrication

The microfluidic DYLC chip was fabricated using a standard PDMS-based soft-lithography process. First, an SU-8 (SU-8 3010, MicroChem Co., USA) master was patterned directly onto a 4-inch silicon wafer using photolithography. The master was then silanized with trichloro (1 H,1 H,2 H,2 H-perfluorooctyl) silane (Sigma-Aldrich Co., USA) in vapor phase to prevent PDMS from sticking to SU-8 surface in replication. Afterwards, a mixture of PDMS oligomer and cross-linking polymer with a weight ratio of 10:1 was degassed and poured onto the SU-8 master, and was cured by baking on a hotplate (PR 5 SR, Harry Gestigkeit GmbH, Germany) at 90 °C for 2 h. Then, the PDMS replicas with channels and micro structures were peeled off from the master, and cut into pieces, which were then punched with 1-mm-diameter holes as inlets and outlets for fluid perfusion. To finalize the chip fabrication, by activating both PDMS and glass surfaces with an oxygen plasma cleaner (PDC-002-HP, Harrick Plasma, USA), each PDMS sheet was irreversibly bonded onto a glass slide that was previously diced from a 500-µm-thick 4-inch Borofloat 33 glass wafer.

### Experimental setup, protocol and image acquisition

#### Setup

An experimental setup to fix the DYLC chip and fit it in the microscopy stage was referred to our previous work [35] and briefly described here. First, the microfluidic chip was clamped between a customized aluminum (AI) holder and a polymethylmethacrylate (PMMA) cover through screws. Then, the AI holder was placed on the multi-well stage of an inverted confocal microscope (FV3000, Olympus Co., Japan). The inlet of the chip was connected to a glass syringe loaded with the cell-culturing medium via polytetrafluoroethylene (PTFE) tubing (BOLA S 1810-08, Bohlender GmbH, Germany). The glass syringe was initially affixed on a programmable syringe pump (neMESYS, Cetoni GmbH, Germany). The whole microscopy stage, as well as the syringe pump with the filled syringe, were placed in a cage incubator (Okolab S.R.L., Italy) controlling the temperature of 30 °C during cell culturing.

#### Protocol

The experimental protocol including chip sterilization, cell loading, trapping, long-term culturing and time-lapse imaging is detailed as follows. To sterilize and clean up the microfluidic DYLC chip, 75% ethanol was firstly perfused over the microchannel at a flow rate of 10 µL/min for 5 min, followed by baking the chip on a hotplate at 100 °C for residual liquid evaporation. Before loading the cell suspension, fresh cell-culturing medium was perfused over the chip at a flow rate of 10 µL/min for 5 min. Afterwards, the chip inlet was switched to a disposable syringe filled with yeast cell suspension, which was infused into the microchannel at a flow rate of 5 µL/min to load cells in the trap array. Once about 70% traps were docked with single yeast cells, the inlet was switched back to the fresh cell-culturing medium, which was continuously supplied throughout the whole experiment at a flow rate of 10 µL/min to maintain a constant hydraulic pressure on immobilized yeast cells for reliable mother retention and effective daughter dissection during long-term cell culturing.

#### Image acquisition

In order to monitor the dynamic processes of cell trapping and rotation, a CMOS camera (E3ISPM, Hangzhou ToupTek Photonics Co., China), inserted into one of the eyepieces through an optical adaptor on the inverted confocal microscope, was used to record videos. Correction ring of the employed objective lens (LUCPLFLN 60×, 0.7NA, Olympus Co., Japan) was adjusted to 0.5 mm to match the thickness of chip substrate.

In order to optimize the cell trapping, maintenance, and hydrodynamic rotation, and perform long-term cell culturing, time-lapse bright-field images were taken at a 10-minute interval by the inverted confocal microscope with a 20× objective lens (UCPLFLN, 0.7NA, Olympus Co., Japan, correction ring adjusted to 0.5 mm) and a cooled GaAsP photomultiplier tube (Olympus Co., Japan). Typically, the whole array divided into six fields were automatically scanned at each time point using the software workflow (FV31S-SW, Olympus Co., Japan). The Z-axis Drift Compensation (ZDC) system (IX3-ZDC2, Olympus Co., Japan) allowed for the sharp focus of samples throughout the whole imaging period.

### Cell preparation

Diploid budding yeast (*Saccharomyces cerevisiae*, strain BY4743 was employed in this work. Dextrose complete (SD) medium was used for cell culturing by standard methods as described in the literature [19]. Solid SD plates (pH 6.5) are composed of 0.17% w/v yeast nitrogen base (YNB) without amino acids, 2% w/v glucose, a complement of amino acids and nucleotides and 15% w/v agar. Liquid SD medium (pH 6.0) has the same compositions as solid SD medium except agar, and the concentration of each composition in liquid SD is the same as that in solid SD medium. Additionally, 0.05% w/v Pluronic^®^ F127 is added in liquid SD to prevent hydrophobic compositions from sticking together or to PDMS traps. All chemicals were purchased from Sigma-Aldrich Co., China.

For cell culturing, yeast cells were first grown on solid SD plates. To prepare cell suspension, yeast was scraped up with a sterile inoculating loop and suspended in liquid SD medium. Suspended yeast cells were then incubated in a flask at 30 ℃ with shaking at 300 rpm for 12 h until cells growing in stationary phase. Afterwards, 100 µL cell suspension was inoculated into another flask containing 10 mL fresh liquid SD medium and incubated for 6 h to resume exponential growth. Before loading cells into the DYLC chip, cell suspension was diluted to approximately 1 × 10^6^ cells per mL.

### Computational fluid dynamics (CFD) modeling and simulation

To optimize the cell-trapping performance in the array, computational fluid dynamics (CFD) simulation in COMSOL Multiphysics software (COMSOL Inc., Burlington, MA, USA) was performed under different geometric conditions, including the distance between adjacent rows (*d*_*r*_: 30 μm, 30 and 34 μm, respectively) and the misalignment in rows (*d*_*m*_: 10 μm, 15 and 17 μm, respectively). To simplify the simulation, the geometric model was established in a 5 × 5 cell-trap array (Additional file 1: Fig. S3A, Table S2). Geometric structures and dimensions of the model were derived from the microfluidic DYLC chip (cf. Fig. 1D). Settings of density of the fluid subdomain, dynamic viscosity, and boundary conditions in numerical simulation are listed in Table S3. Specially, to amplify the subtle differences between different array settings, the laminar flow speed at the entrance was set larger than the equivalent value from the medium perfusion conditions used in practice. Contour plots referring to the flow velocity distribution were used to investigate the fluid field in the 5 × 5 array with different settings of *d*_*r*_ and *d*_*m*_. Based on the obtained fluid field in the middle cross-section of the array (Additional file 1: Fig. S3B), time-dependent particle trajectory tracing was calculated to study the dynamic process of cell trapping (Additional file 1: Table S4). In addition, for better illustration of cell trajectories before and after cell capture in traps, two columns of cell traps upstream were set empty and filled with single cells, respectively.

To investigate the bud rotation and concerted daughter-cell dissection, hydrodynamic forces over a budding yeast cell immobilized in a single trap were calculated [36]. The budding yeast was modeled as a conjugation of an ellipsoidal mother (5 μm × 4.4 μm) and a spheroidal bud (2 μm in diameter), contacting with each other at the upstream end of the major axis of the ellipsoid (Additional file 1: Fig. S4). To imitate the bud rotation in the trap, the mother cell together with its bud, were set to rotate from -x to + x direction along the xz-plane with a 45-degree interval, meanwhile the mother was kept contacting with the “bowl” bottom. To imitate bud growth after the bud was relocated in the narrow orifice, the diameter of the sphere was set 2 μm, 3 and 4 μm, respectively. Hydrodynamic forces over the bud of each setting of orientation and diameter were numerically calculated. Arrows with quantified lengths were labeled on the cell to visualize the force values in both 3D and planar projection views.

### Data analysis of RLS and budding time interval

**Fig. 7:**
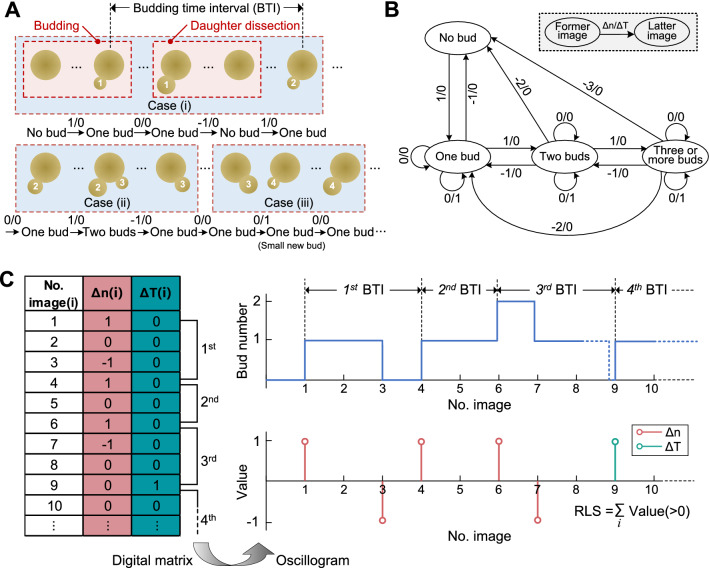
Data analysis principle and processes of yeast RLS and budding time interval. **A** Three representative cases of cell budding and dissection events between two adjacent buds. Two-digit codes below cells marked the change of bud number. **B** Using the two-digit codes to enumerate the orders of bud appearance and daughter dissection in experiments. **C** Intuitive calculation of RLS and budding time interval by using the digital matrix and oscillogram derived from **A**

To calculate yeast RLS and budding time interval from time-lapse images, cell events, including budding and daughter dissection, were analyzed by comparing the changes of bud number between two adjacent images. In experiment, sometimes cell events did not occur in order, or could not be recorded within the 10-minute time interval of imaging, thereby resulting in three representative cases (Fig. 7A). (i) Former bud was observed from its appearance to removal, after which came the latter bud orderly with an observable time gap. At least one image could show that the mother cell held no bud after the removal of the first bud and before the appearance of the second bud. (ii) Latter bud sprouted before the removal of former bud. Images in this case could show that the mother cell held two buds in a certain time period. (iii) The removal of former bud and the appearance of latter bud occurred orderly without an observable time gap. In this case, a big bud could be observed in an image, but in the next image it disappeared and meanwhile a small bud was observed.

To efficiently analyze cell events of yeast budding and daughter dissection of each immobilized mother cell, sequential two-digit coding, which corresponded to each frame of time-lapse images, was devised to mark the change of bud number (‘no bud’, ‘one bud’, ‘two buds’ and ‘three or more buds’) (Fig. 7B). Each code, comprising ‘Δn’ and ‘ΔT’, was defined as follows: ‘Δn’ denoted the change of bud number between two adjacent images; ‘0’, ‘1’ and ‘-1’ of ‘Δn’ indicated no change in bud number, production of a new bud and removal of an old bud, respectively. ‘ΔT’ marked whether there was an observable time gap in images showing the mother holding no bud for clearly separating two adjacent generations. For instance, ‘ΔT’ = ‘1’ indicated that the removal of former bud and the appearance of a new bud occurred within a 10-minute interval (cf. Case (iii) in Fig. 7A). Then, the sequential codes of the entire time-lapse images of a recorded cell were obtained to enumerate all budding and dissection events of a recorded cell and transformed into a digital matrix (Fig. 7C). Consequently, the yeast RLS was determined by summating the total number of ‘1’ in ‘Δn’ and ‘ΔT’. Additionally, the budding time interval, which was defined as the time duration between two adjacent successive budding events, could be calculated by the following steps: (1) subtracting the former image number, of which ‘Δn(i)’ or ‘ΔT(i)’ were marked as ‘1’, from the latter one; (2) multiplying the difference from last step by 10 min. Then, the budding time intervals throughout the whole lifespan of a recorded cell could be obtained as a function of the generation of buds. Finally, an oscillogram developed from the digital matrix could intuitively illustrate the calculation processes of RLS and budding time intervals (Fig. 7C).

### Data statistics

In data statistics, standard error (SE), which was calculated by dividing the square root of independent runs into the standard deviation, was used to evaluate the precision between the sample mean and the population mean unless stated separately.

## Supplementary Information

**Additional file 1**:** Figure S1**. CFD simulation of hydrodynamic forces exerted on an immobilized budding yeast cell. (A) Hydrodynamic forces on the bud quantified by arrow length during hydrodynamic rotation of budding yeast from upstream to downstream in a 45-degree interval on the xz plane. (B) Hydrodynamic forces on a growing bud (diameter: 2 μm, 3 μm and 4 μm) towards the downstream to evaluate the cell retention and daughter detachment in the orifice.** Figure S2** Calculation of RLS and BTI by using the digital matrix and oscillogram recorded from the representative cell in Figure 5(A).** Figure S3** Wireframes of the model geometry containing channel walls (black), a 5 × 5 array of cell traps (blue) and immobilized yeast cells (brown) and culturing medium filled in the residual space of the model. (A) 3D view of the simplified geometric model established as follows: A 5 × 5 cell-trap array with different settings of geometric features (dr and dm) was placed in the middle of a microchannel, which featured 400 μm × 8 μm outer profile in xz-dimension and variable length dy in y-axis for fitting 5 columns of traps. The distance between adjacent columns was set to 30 μm. (B) 2D cross-section view of the array for particle trajectory tracing. dr, dm and dy in modeling were set to 34 μm, 17 μm and 158 μm, respectively.** Figure S4**. 3D wireframe of the model geometry in simulation of bud rotation in a single trap, containing channel walls (black), a trap (blue) and a budding yeast cell (brown) and culturing medium filled in the residual space of the model. Geometric conditions were set as follows: The trap was placed in the center with 30 μm × 34 μm × 8 μm outer profile in xyz-dimension; the budding yeast cell was set as a conjugation of an ellipsoid (5 μm × 4.4 μm) and a sphere (2 μm in diameter), contacting with each other at the upstream end of the major axis of the ellipsoid initially. To imitate the bud rotation in the trap, the mother cell together with its bud, were set to rotate from -x to +x direction along the xz-plane with a 45-degree interval, meanwhile the mother was kept contacting with the “bowl” bottom; to imitate bud growth after the bud was relocated in the narrow orifice, the diameter of the sphere was set 2 μm, 3 μm and 4 μm, respectively.** Table S1**. A comparison of reported RLS data of budding yeast using conventional and microfluidic methods.** Table S2**. Geometric settings of the cell-trap array in CFD simulation.** Table S3**. General settings and parameters in CFD simulation.** Table S4**. Settings and parameters of particle trajectory tracing in cell-trapping simulation

**Additional file 2**:** Video S1** Video of CFD simulation showing the dynamic process of cell trapping in the array. Cells in suspension bypass the occupied traps and are captured in empty traps downstream.

**Additional file 3**:** Video S2** A budded yeast cell was rotating when flowing towards an empty trap, and then immobilized at the trap. Afterwards, cells coming upstream were bypassing the occupied trap. In order to record the cell movement, the flow rate was slowed down to 0.5 μL/min. Scale bar is 10 μm.

**Additional file 4**:** Video S3** Bud rotating, swaying and fixedness of immobilized budding yeast cells with their buds at different sizes. Scale bar is 5 μm.

**Additional file 5**:** Video S4** Momentary daughter dissection and immediate bud reorientation towards downstream. Scale bar is 5 μm.

**Additional file 6**:** Video S5** Time-lapse images of the whole lifespan of an immobilized budding yeast cell from newborn to death (RLS: 25 generations). Scale bar is 5 μm.

## Data Availability

All data used to support the findings of this study are included within the article.

## References

[1] Mortimer RK, Johnston JR (1959). Life span of individual yeast cells. Nature.

[2] Botstein D, Fink GR (2011). Yeast: an experimental organism for 21st century biology. Genetics.

[3] Feldmann H (2010). Yeast: molecular and cell biology.

[4] Tenreiro S, Outeiro TF (2010). Simple is good: yeast models of neurodegeneration. FEMS Yeast Res.

[5] Pearce DA, Sherman F (1998). A yeast model for the study of Batten disease. Proc Natl Acad Sci USA.

[6] Coelho MC, Pinto RM, Murray AW (2019). Heterozygous mutations cause genetic instability in a yeast model of cancer evolution. Nature.

[7] Weinberger M, Feng L, Paul A, Smith DL, Hontz RD, Smith JS, Vujcic M, Singh KK, Huberman JA, Burhans WC (2007). DNA replication stress is a determinant of chronological lifespan in budding yeast. PLoS ONE.

[8] Novarina D, Janssens GE, Bokern K, Schut T, van Oerle NC, Kazemier HG, Veenhoff LM, Chang M (2020). A genome-wide screen identifies genes that suppress the accumulation of spontaneous mutations in young and aged yeast cells. Aging Cell.

[9] Gershon H, Gershon D (2000). The budding yeast, *Saccharomyces cerevisiae*, as a model for aging research: a critical review. Mech Ageing Dev.

[10] Raser JM (2004). Control of stochasticity in eukaryotic gene expression. Science.

[11] Longo VD, Shadel GS, Kaeberlein M, Kennedy B (2012). Replicative and chronological aging in* Saccharomyces cerevisiae*. Cell Metab.

[12] Lee SS, Avalos Vizcarra I, Huberts DH, Lee LP, Heinemann M (2012). Whole lifespan microscopic observation of budding yeast aging through a microfluidic dissection platform. Proc Natl Acad Sci USA.

[13] Zhang Y, Luo C, Zou K, Xie Z, Brandman O, Ouyang Q, Li H (2012). Single cell analysis of yeast replicative aging using a new generation of microfluidic device. PLoS ONE.

[14] Crane MM, Clark IB, Bakker E, Smith S, Swain PS (2014). A microfluidic system for studying ageing and dynamic single-cell responses in budding yeast. PLoS ONE.

[15] Jo MC, Liu W, Gu L, Dang W, Qin L (2015). High-throughput analysis of yeast replicative aging using a microfluidic system. Proc Natl Acad Sci USA.

[16] Liu P, Young TZ, Acar M (2015). Yeast replicator: a high-throughput multiplexed microfluidics platform for automated measurements of single-cell aging. Cell Rep.

[17] Ryley J, Pereira-Smith OM (2006). Microfluidics device for single cell gene expression analysis in *Saccharomyces cerevisiae*. Yeast.

[18] Sarnoski EA, Song R, Ertekin E, Koonce N, Acar M (2018). Fundamental characteristics of single-cell aging in diploid yeast. iScience.

[19] Sherman F (1991). Getting started with yeast. Methods Enzymol.

[20] Chant J, Pringle JR (1995). Pringle patterns of bud-site selection in the yeast *Saccharomyces cerevisiae*. J Cell Biol.

[21] Wang Y, Lo WC, Chou CS (2017). A modeling study of budding yeast colony formation and its relationship to budding pattern and aging. PLoS Comput Biol.

[22] Jazwinski SM, Wawryn J (2001). Profiles of random change during aging contain hidden information about longevity and the aging process. J Theor Biol.

[23] Geng Y, Zhu Z, Wang Y, Wang Y, Ouyang S, Zheng K, Ye W, Fan Y, Wang Z, Pan D (2019). Multiplexing microelectrodes for dielectrophoretic manipulation and electrical impedance measurement of single particles and cells in a microfluidic device. Electrophoresis.

[24] Zhu Z, Wang Y, Peng R, Chen P, Geng Y, He B, Ouyang S, Zheng K, Fan Y, Pan D, Jin N, Rudolf F, Hierlemann A (2021). A microfluidic single-cell array for in situ laminar-flow-based comparative culturing of budding yeast cells. Talanta.

[25] Kennedy BK, Austriaco NR, Guarente L (1994). Daughter cells of *Saccharomyces cerevisiae* from old mothers display a reduced life span. J Cell Biol.

[26] Yang J, McCormick MA, Zheng J, Xie Z, Tsuchiya M, Tsuchiyama S, El-Samad H, Ouyang Q, Kaeberlein M, Kennedy BK, Li H (2015). Systematic analysis of asymmetric partitioning of yeast proteome between mother and daughter cells reveals “aging factors” and mechanism of lifespan asymmetry. Proc Natl Acad Sci USA.

[27] Ferrezuelo F, Colomina N, Palmisano A, Garí E, Gallego C, Csikász-Nagy A, Aldea M (2012). The critical size is set at a single-cell level by growth rate to attain homeostasis and adaptation. Nat Commun.

[28] Lv C, Li X, Li F, Li T (2015). Energy landscape reveals that the budding yeast cell cycle is a robust and adaptive multi-stage process. PLoS Comput Biol.

[29] Yang J, Dungrawala H, Hui H, Manukyan A, Abraham L, Lane W, Mead H, Wright J, Schneider BL (2011). Cell size and growthrate are major determinants of replicative lifespan. Cell Cycle.

[30] Qin H, Lu M (2006). Natural variation in replicative and chronological life spans of *Saccharomyces cerevisiae*. Exp Gerontol.

[31] Minois N, Frajnt M, Wilson C, Vaupel JW (2005). Advances in measuring lifespan in the yeast *Saccharomyces cerevisiae*. Proc Natl Acad Sci USA.

[32] Gao Z, Xu J, Chen K, Wang S, Ouyang Q, Luo C (2020). Comparative analysis of yeast replicative lifespan in different trapping structures using an integrated microfluidic system. Adv Mater Technol.

[33] Mcmurray MA, Gottschling DE (2005). Aging and genetic instability in yeast. Curr Opin Microbiol.

[34] Aström SU, Okamura SM, Rine J (1999). Yeast cell-type regulation of DNA repair. Nature.

[35] Zhu Z, Frey O, Haandbaek N, Franke F, Rudolf F, Hierlemann A (2015). Time-lapse electrical impedance spectroscopy for monitoring the cell cycle of single immobilized *S. pombe* cells. Sci Rep.

[36] Xu X, Zhu Z, Wang Y, Geng Y, Xu F, Marchisio MA, Wang Z, Pan D (2021). Investigation of daughter cell dissection coincidence of single budding yeast cells immobilized in microfluidic traps. Anal Bioanal Chem.

