# Supplementary material for: A high-throughput microfluidic diploid yeast long-term culturing (DYLC) chip capable of bud reorientation and concerted daughter dissection for replicative lifespan determination
Source: J Nanobiotechnology. 2022 Mar 31;20:171. doi: 10.1186/s12951-022-01379-9 (PMC8973578; doi:10.1186/s12951-022-01379-9)
Supplement: Supplementary file 1 — Additional file 1: Figure S1. CFD simulation of hydrodynamic forces exerted on an immobilized budding yeast cell. (A) Hydrodynamic forces on the bud quantified by arrow length during hydrodynamic rotation of budding yeast from upstream to downstream in a 45-degree interval on the xz plane. (B) Hydrodynamic forces on a growing bud (diameter: 2 μm, 3 μm and 4 μm) towards the downstream to evaluate the cell retention and daughter detachment in the orifice. Figure S2 Calculation of RLS and BTI by using the digital matrix and oscillogram recorded from the representative cell in Figure 5(A). Figure S3 Wireframes of the model geometry containing channel walls (black), a 5 × 5 array of cell traps (blue) and immobilized yeast cells (brown) and culturing medium filled in the residual space of the model. (A) 3D view of the simplified geometric model established as follows: A 5 × 5 cell-trap array with different settings of geometric features (dr and dm) was placed in the middle of a microchannel, which featured 400 μm × 8 μm outer profile in xz-dimension and variable length dy in y-axis for fitting 5 columns of traps. The distance between adjacent columns was set to 30 μm. (B) 2D cross-section view of the array for particle trajectory tracing. dr, dm and dy in modeling were set to 34 μm, 17 μm and 158 μm, respectively. Figure S4. 3D wireframe of the model geometry in simulation of bud rotation in a single trap, containing channel walls (black), a trap (blue) and a budding yeast cell (brown) and culturing medium filled in the residual space of the model. Geometric conditions were set as follows: The trap was placed in the center with 30 μm × 34 μm × 8 μm outer profile in xyz-dimension; the budding yeast cell was set as a conjugation of an ellipsoid (5 μm × 4.4 μm) and a sphere (2 μm in diameter), contacting with each other at the upstream end of the major axis of the ellipsoid initially. To imitate the bud rotation in the trap, the mother cell together with its bud, were set to [file 12951_2022_1379_MOESM1_ESM.pdf]

A high-throughput microfluidic diploid yeast long-term culturing (DYLC) chip capable of bud reorientation and concerted daughter dissection for replicative lifespan determination

Yingying Wang[‡a], Zhen Zhu[‡*a], Ke Liu[a], Qin Xiao[a], Yangye Geng[a], Feng Xu[a], Shuiping Ouyang[b], Ke Zheng[b], Yimin Fan[b], Nan Jin[c], Xiangwei Zhao[d], Mario A. Marchisio[e], Dejing Pan[f], and Qing-an Huang[a]

[a] Southeast University, Key Laboratory of MEMS of Ministry of Education, Sipailou 2, Nanjing 210096, China

[b] Nanjing Forestry University, College of Chemical Engineering, Longpan Road 159, Nanjing 210037, China

[c] Southeast University, ZhongDa Hospital, Dingjiaqiao 87, Nanjing 210009, China

[d] Southeast University, State Key Laboratory of Bioelectronics, Sipailou 2, Nanjing 210096, China

[e] Tianjin University, School of Pharmaceutical Science and Technology, Weijin Road 92, Tianjin 300072, China

[f] Soochow University, Cambridge-Suda Genomic Resource Center and Jiangsu Key Laboratory of Neuropsychiatric Diseases Research,, Ren-ai Road 199, Suzhou 215213, China

[‡] Equal contribution

[*] To whom correspondence may be addressed. Email: zhuzhen@seu.edu.cn

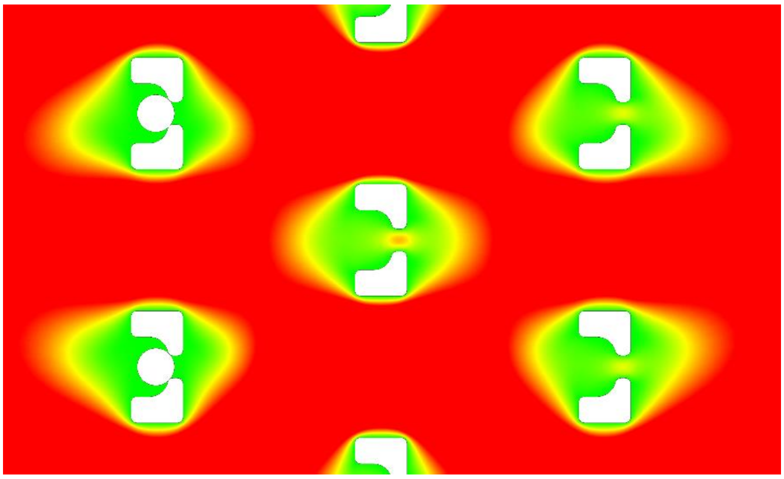

Supplementary Video S1 Video of CFD simulation showing the dynamic process of cell trapping in the array. Cells in suspension bypass the occupied traps and are captured in empty traps downstream.

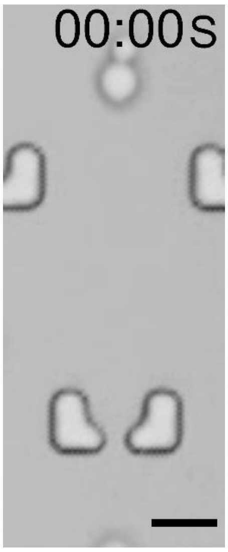

Supplementary Video S2 A budded yeast cell was rotating when flowing towards an empty trap, and then immobilized at the trap. Afterwards, cells coming upstream were bypassing the occupied trap. In order to record the cell movement, the flow rate was slowed down to 0.5 μL/min. Scale bar is 10 μm.

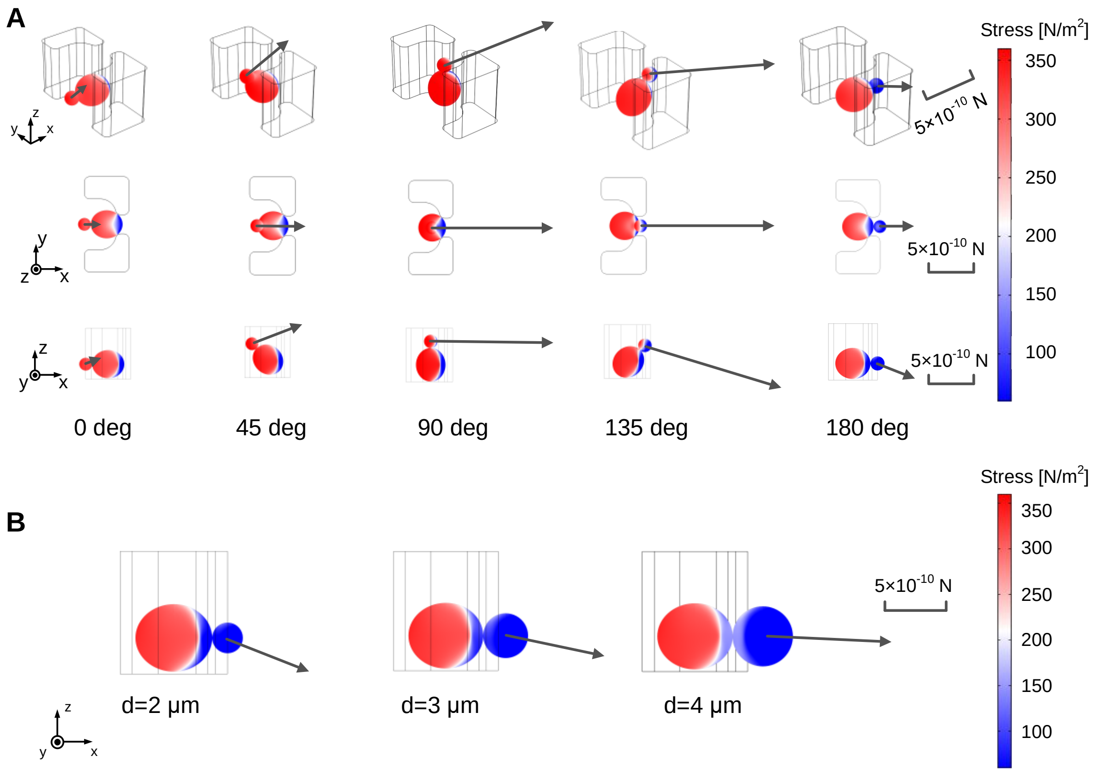

Supplementary Figure S1 CFD simulation of hydrodynamic forces exerted on an immobilized budding yeast cell. (A) Hydrodynamic forces on the bud quantified by arrow length during hydrodynamic rotation of budding yeast from upstream to downstream in a 45-degree interval on the xz plane. (B) Hydrodynamic forces on a growing bud (diameter: 2 μm, 3 μm and 4 μm) towards the downstream to evaluate the cell retention and daughter detachment in the orifice.

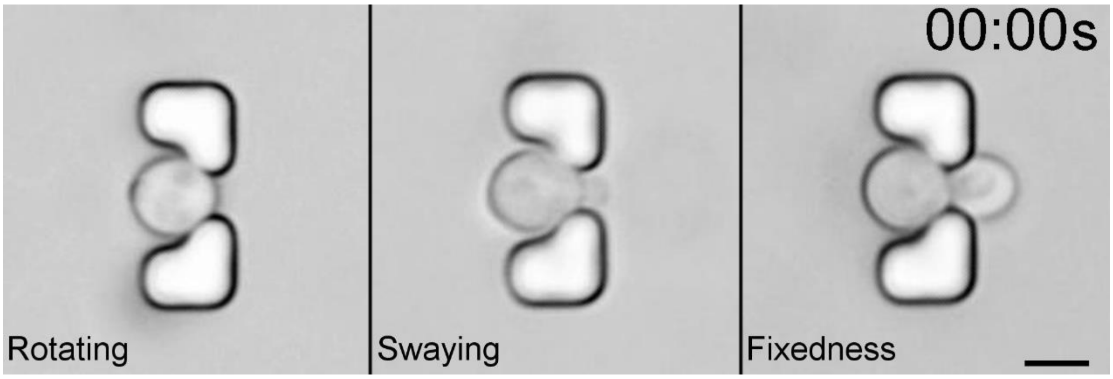

Supplementary Video S3 Bud rotating, swaying and fixedness of immobilized budding yeast cells with their buds at different sizes. Scale bar is 5 μm.

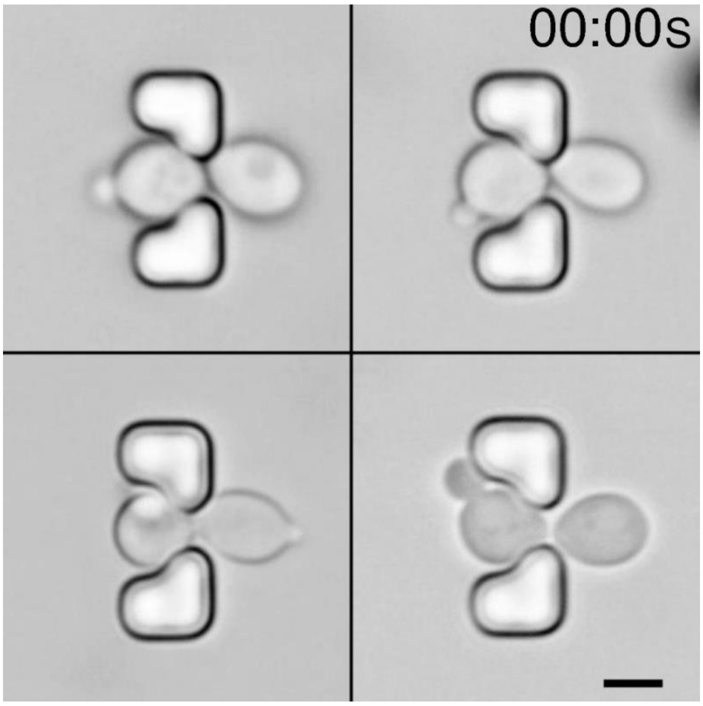

Supplementary Video S4 Momentary daughter dissection and immediate bud reorientation towards downstream. Scale bar is 5 μm.

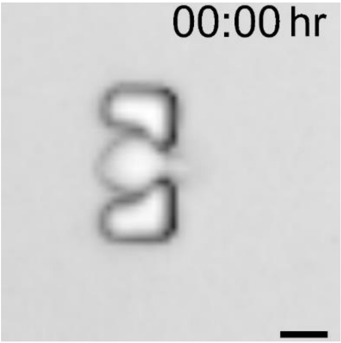

Supplementary Video S5 Time-lapse images of the whole lifespan of an immobilized budding yeast cell from newborn to death (RLS: 25 generations). Scale bar is 5 μm.

A

No. image(i)	Δn(i)	ΔT(i)	Event(s)
0			
3	1	0	Bud appearing
8	0	0	Bud growing
9	-1	0	Bud dissected
11	1	0	Budding
17	0	0	Bud growing
18	1	0	New bud appearing
20	-1	0	Former bud dissected
24	0	0	Bud growing
25	0	1	Bud dissected & new bud appearing
34	1	0	New bud appearing
38	-1	0	Former bud dissected
39	-1	0	Latter bud dissected
41	1	0	Bud appearing
47	0	0	Bud growing
49	0	1	Bud dissected & new bud appearing
53	0	0	Bud growing
55	0	1	Bud dissected & new bud appearing
60	0	0	Bud growing
62	0	1	Bud dissected & new bud appearing
66	0	0	Bud growing
67	-1	0	Bud dissected
69	1	0	Bud appearing
74	0	0	Bud growing
76	0	1	Bud dissected & new bud appearing
83	1	0	New bud appearing
89	1	0	New bud appearing
90	-1	0	Latter bud dissected
92	-1	0	Former bud dissected
94	-1	0	Latter bud dissected

No. image(i)	Δn(i)	ΔT(i)	Event(s)
96	1	0	Bud appearing
100	0	0	Bud growing
101	-1	0	Bud dissected
103	1	0	Bud appearing
107	0	0	Bud growing
108	-1	0	Bud dissected
110	1	0	Bud appearing
114	0	0	Bud growing
115	-1	0	Bud dissected
117	1	0	Bud appearing
122	0	0	Bud growing
124	1	0	New bud appearing
125	-1	0	Former bud dissected
130	0	0	Bud growing
132	1	0	New bud appearing
133	-1	0	Former bud dissected
138	0	0	Bud growing
140	1	0	New bud appearing
145	0	0	Bud growing
148	-1	0	Latter bud dissected
150	1	0	New bud appearing
152	-1	0	Former bud dissected
158	-1	0	Latter bud dissected
160	1	0	Bud appearing
168	0	0	Bud growing
169	-1	0	Bud dissected
174	1	0	Bud appearing
186	0	0	Bud growing
187	-1	0	Bud dissected
198			Dead

Digital matrix

Oscillogram

B

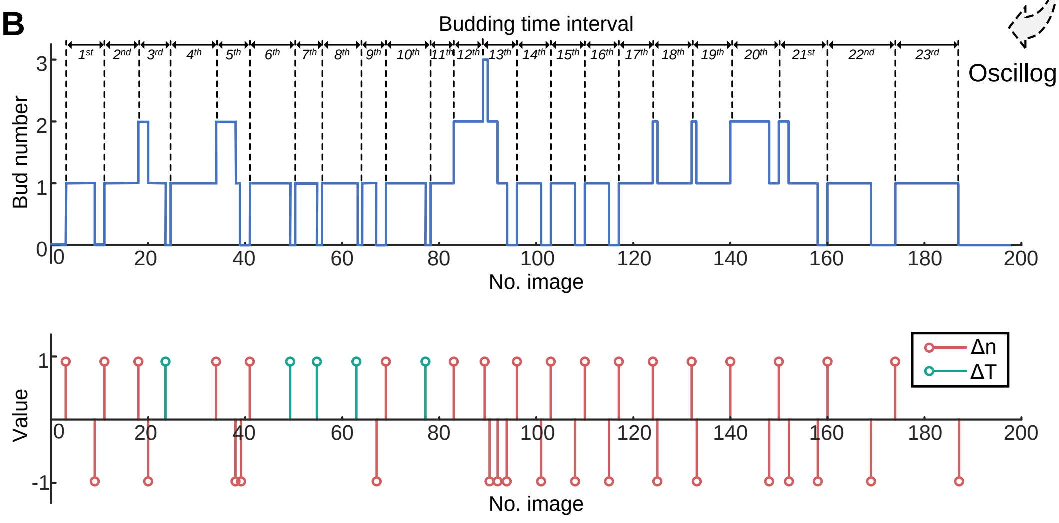

Supplementary Figure S2 Calculation of RLS and BTI by using the digital matrix and oscillogram recorded from the representative cell in Figure 5(A).

Supplementary Table S1. A comparison of reported RLS data of budding yeast using conventional and microfluidic methods.

Reference	Strain	RLS	Methods for daughter cell removal	No. samples
This paper [a]	diploid BY4743	24.29±3.65	Microfluidic	786
[1] Sarnoski, iScience, 2018 [b]	diploid BY4743	29±0.7	Microfluidic	50
[2] Yang, Cell Cycle, 2011	diploid BY4743	26.5	Manual	30-50
[3] Lee, PNAS, 2019	diploid AH2601 AH2801	24	Manual	161
[4] Kaeberlein, Ageing Dev., 2005	diploid BY4743	37.5	Manual	110
[5] Qin, Exp. Gerontol., 2006	diploid BY4743	33.2±0.9	Manual	90
[6] Delaney, FEMS Yeast Res., 2013	diploid BY4743	33.7	Manual	529
[7] Jo, PNAS, 2015 [c]	haploid BY4741	25.76	Microfluidic	458
[8] Crane, PLOS ONE, 2014 [d]	haploid S288C	22.4	Microfluidic	422
[9] Lee, PNAS, 2012 [e]	haploid S288C	21	Microfluidic	76
[10] Liu, Cell Rep., 2015 [f]	haploid BY background	29.3	Microfluidic	100
[11] Minois, PNAS, 2015	haploid S288C	29.96±1.72	Manual	46
[12] Defossez, Mol. Cell, 1999	haploid K2307	23.7	Manual	50
	haploid W303	21.6		37
[13] Kang, Mol. Biol. Cell, 2022	haploid BY4741	29	Manual	28
[14] Smith, Genome Res., 2008	haploid BY4742	24.4	Manual	20

Separately, the microstructures designed for accommodating budding yeast cells in the microfluidic methods were [a] "leaky bowl"-shaped traps, [b] "[ ]" -shaped cages, [c] "U"-shaped traps, [d] "/ \"-shape traps, [e] "pensile pads", [f] "U"-shaped traps.

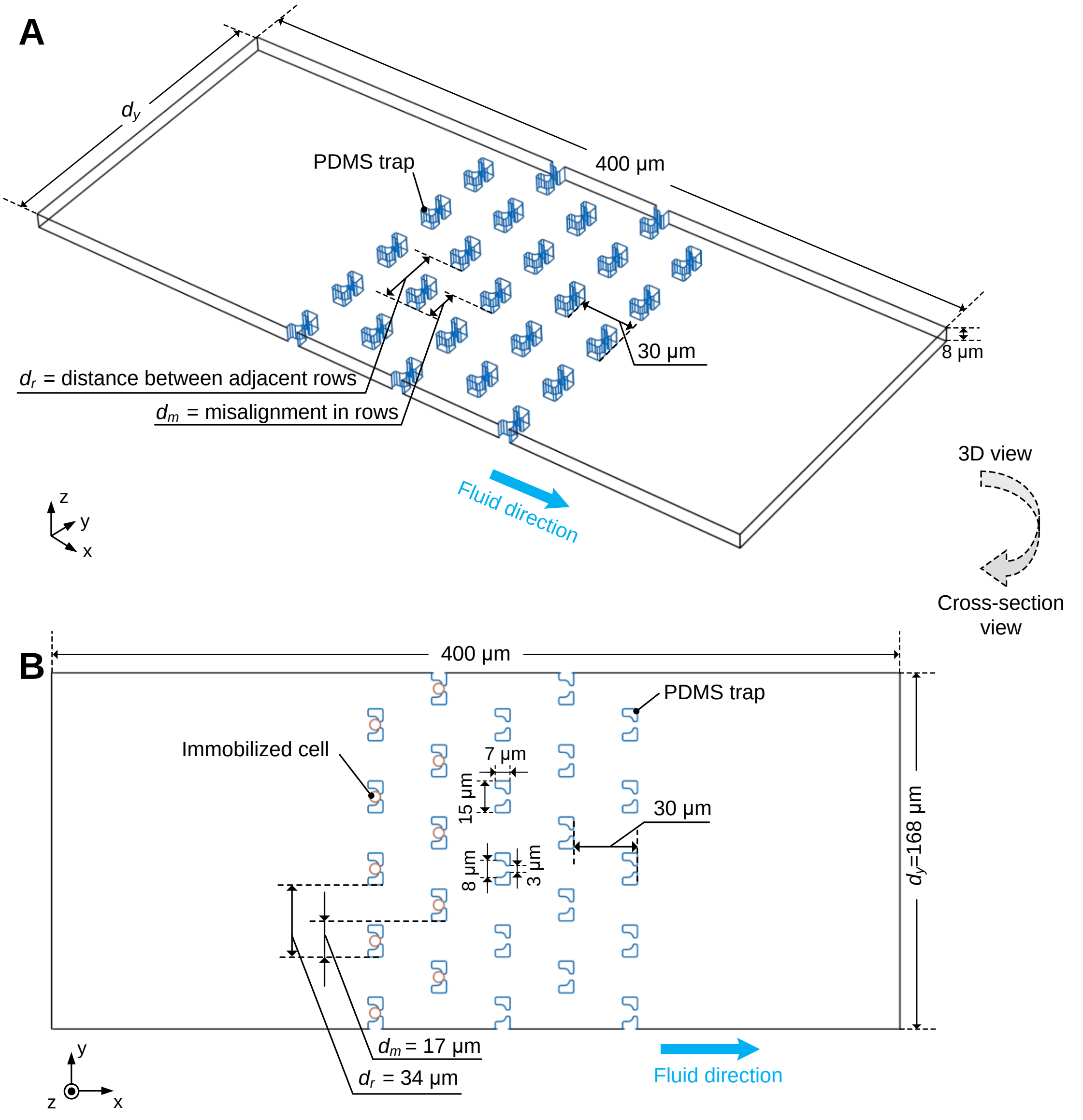

Supplementary Figure S3 Wireframes of the model geometry containing channel walls (black), a 5 × 5 array of cell traps (blue) and immobilized yeast cells (brown) and culturing medium filled in the residual space of the model. (A) 3D view of the simplified geometric model established as follows: A 5 × 5 cell-trap array with different settings of geometric features (d_r and d_m) was placed in the middle of a microchannel, which featured 400 μm × 8 μm outer profile in xz-dimension and variable length d_y in y-axis for fitting 5 columns of traps. The distance between adjacent columns was set to 30 μm. (B) 2D cross-section view of the array for particle trajectory tracing. d_r, d_m and d_y in modeling were set to 34 μm, 17 μm and 158 μm, respectively.

Supplementary Table S2. Geometric settings of the cell-trap array in CFD simulation

Setting	Distance between adjacent rows (d_r) [µm]	Misalignment in rows (d_m) [µm]	Outer profile in y-dimension (d_y) [µm]
1	30	10 ($\frac{1}{3}d_r$)	155
2	30	15 ($\frac{1}{2}d_r$)	150
3	34	17 ($\frac{1}{2}d_r$)	168

Supplementary Table S3. General settings and parameters in CFD simulation

Parameter	Setting/Value
Boundary condition at walls	No-slip condition
Flow speed at inlet	5 m/s
Density of fluid subdomain	1000 kg/m^3
Dynamic viscosity	0.001 Pa•s

Supplementary Table S4. Settings and parameters of particle trajectory tracing in cell-trapping simulation

Parameter		Setting/Value
Flow speed at inlet		0.004 m/s
Boundary condition of particles at walls		Bounce
Particle properties	Mass	1×10^{-12} kg
	Diameter	1×10^{-6} m
Inlet boundary	Particle initial position	Uniform distribution
	Number of particles	200
	Particle initial velocity	Equal to fluid velocity
	Particle release time	0 s

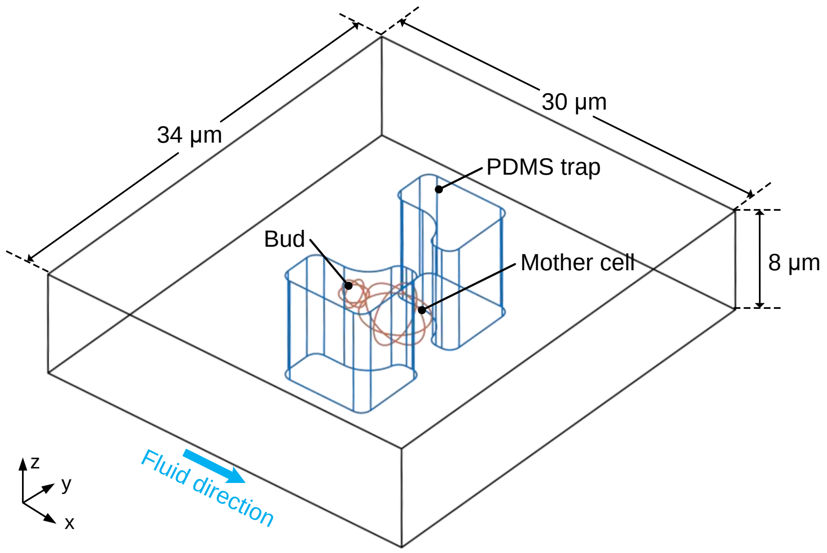

Supplementary Figure S4 3D wireframe of the model geometry in simulation of bud rotation in a single trap, containing channel walls (black), a trap (blue) and a budding yeast cell (brown) and culturing medium filled in the residual space of the model. Geometric conditions were set as follows: The trap was placed in the center with 30 μm × 34 μm × 8 μm outer profile in xyz-dimension; the budding yeast cell was set as a conjugation of an ellipsoid (5 μm × 4.4 μm) and a sphere (2 μm in diameter), contacting with each other at the upstream end of the major axis of the ellipsoid initially. To imitate the bud rotation in the trap, the mother cell together with its bud, were set to rotate from -x to +x direction along the xz-plane with a 45-degree interval, meanwhile the mother was kept contacting with the “bowl” bottom; to imitate bud growth after the bud was relocated in the narrow orifice, the diameter of the sphere was set 2 μm, 3 μm and 4 μm, respectively.

Reference

[1] Sarnoski EA, Song R, Ertekin E, Koonce N, Acar M (2018) Fundamental Characteristics of Single-Cell Aging in Diploid Yeast. iScience 7, 96-109. 10.1016/j.isci.2018.08.011.

[2] Yang J, Dungrawala H, Hua H, Manukyan A, Abraham L, Lane W, Mead H, Wright J, Schneider BL (2011). Cell size and growth rate are major determinants of replicative lifespan. Cell cycle 10(1): 144-155. org/10.4161/cc.10.1.14455

[3] Lee MB, Dowsett IT, Carr DT, Wasko BM, Stanton SG, Chung MS, Ghodsian N, Bode A, Kiflezghi MG, Uppal PA, Grayden KA, Elala YC, Tang TT, Tran NHB, Tran THB, Diep AB, Hope M, Promislow DEL, Kennedy SR, Kaeberlein M, Herr AJ (2019) Defining the impact of mutation accumulation on replicative lifespan in yeast using cancer-associated mutator phenotypes. Proc Natl Acad Sci U S A., 116(8): 3062-3071.

[4] Kaeberlein M, Kirkland KT, Fields S, et al (2015) Genes determining yeast replicative life span in a long-lived genetic background. Mech. Ageing Dev. 126(4):491-504.

[5] Qin H, Lu M (2006) Natural variation in replicative and chronological life spans of Saccharomyces cerevisiae. Exp. Gerontol. 41(4), 448-456. 10.1016/j.exger.2006.01.007.

[6] Delaney J R, Chou A, Olsen B, et al (2013) End-of-life cell cycle arrest contributes to stochasticity of yeast replicative aging. FEMS Yeast Res. 13(3): 267-276.

[7] Jo MC, Liu W, Gu L, Dang W, Qin L (2015) High-throughput analysis of yeast replicative aging using a microfluidic system. Proc Natl Acad Sci U S A. 112, 9364-9369. 10.1073/pnas.1510328112.

[8] Crane MM, Clark IB, Bakker E, Smith S, Swain PS (2014) A Microfluidic System for Studying Ageing and Dynamic Single-Cell Responses in Budding Yeast. PLoS One 9, e100042. 10.1371/journal.pone.0100042.

[9] Lee SS, Avalos Vizcarra I, Huberts DH, Lee LP, Heinemann M (2012) Whole lifespan microscopic observation of budding yeast aging through a microfluidic dissection platform. Proc Natl Acad Sci U S A 109, 4916-4920.

[10] Liu P, Young TZ, Acar M (2015) Yeast Replicator: A High-Throughput Multiplexed Microfluidics Platform for Automated Measurements of Single-Cell Aging. Cell Rep. 13, 634-644. 10.1016/j.celrep.2015.09.012.

[11] Minois N, Frajnt M, Wilson C, Vaupel JW (2005) Advances in measuring lifespan in the yeast Saccharomyces cerevisiae. Proc Natl Acad Sci U S A. 102(2), 402-406.

[12] Defossez PA, Prusty R, Kaeberlein M, et al (1999) Elimination of replication

block protein Fob1 extends the life span of yeast mother cells. Mol. cell, 3(4): 447-455.

[13] Kang P J, Mullner R, Li H, et al (2022) Upregulation of the Cdc42 GTPase limits the replicative lifespan of budding yeast. Mol. Biol. Cell E20-01-0087.

[14] Smith ED, Tsuchiya M, Fox LA, et al (2018) Quantitative evidence for conserved longevity pathways between divergent eukaryotic species. Genome Res. 18(4): 564-570.
